# piRNA‐63076 contributes to pulmonary arterial smooth muscle cell proliferation through acyl‐CoA dehydrogenase

**DOI:** 10.1111/jcmm.15179

**Published:** 2020-03-30

**Authors:** Cui Ma, Lixin Zhang, Xiaoying Wang, Siyu He, June Bai, Qian Li, Min Zhang, Chen Zhang, Xiufeng Yu, Junting Zhang, Wei Xin, Yiying Li, Daling Zhu

**Affiliations:** ^1^ Central Laboratory of Harbin Medical University (Daqing) Daqing China; ^2^ College of Medical Laboratory Science and Technology Harbin Medical University (Daqing) Daqing China; ^3^ College of Pharmacy Harbin Medical University Harbin China; ^4^ College of Pharmacy Harbin University of Commerce Harbin China; ^5^ State Province Key Laboratories of Biomedicine Pharmaceutics of China Daqing China; ^6^ Key Laboratory of Cardiovascular Medicine Research Ministry of Education Harbin Medical University Harbin China

**Keywords:** acyl‐CoA dehydrogenase, hypoxia, piwi‐interacting RNAs, pulmonary hypertension

## Abstract

Piwi‐interacting RNAs (piRNAs) are thought to be germline‐specific and to be involved in maintaining genome stability during development. Recently, piRNA expression has been identified in somatic cells in diverse organisms. However, the roles of piRNAs in pulmonary arterial smooth muscle cell (PASMC) proliferation and the molecular mechanism underlying the hypoxia‐regulated pathological process of pulmonary hypertension are not well understood. Using hypoxic animal models, cell and molecular biology, we obtained the first evidence that the expression of piRNA‐63076 was up‐regulated in hypoxia and was positively correlated with cell proliferation. Subsequently, we showed that acyl‐CoA dehydrogenase (Acadm), which is negatively regulated by piRNA‐63076 and interacts with Piwi proteins, was involved in hypoxic PASMC proliferation. Finally, Acadm inhibition under hypoxia was partly attributed to DNA methylation of the Acadm promoter region mediated by piRNA‐63076. Overall, these findings represent invaluable resources for better understanding the role of epigenetics in pulmonary hypertension associated with piRNAs.

## 1 INTRODUCTION

Sustained hypoxic exposure results in increased pulmonary vascular resistance, pulmonary vascular remodelling and pulmonary hypertension (PH).^1^, ^2^ Pulmonary arterial smooth muscle cells (PASMCs) exhibit significant cellular and structural changes within the media of the pulmonary vasculature including augmented proliferation, resistance to apoptosis, increased cell adhesion and migration and secretion of extracellular matrix proteins.^3^, ^4^ It is necessary to elucidate the molecular and cellular basis underlying the pathogenesis of PH and to develop molecular compounds that reduce excessive PASMC proliferation to treat patients with PH.

Recently, small non‐coding RNA molecules along with the Argonaute family of proteins have been identified as key regulators of biological processes by sequence‐specific gene silencing in various human diseases.^5^, ^6^ In mammals, Argonaute proteins segregate into two subfamilies.^7^ The Argonaute subfamily acts in microRNA‐mediated RNA interference by repressing translation or directing sequence‐specific degradation of target mRNAs.^8^ The Piwi subfamily acts in germline stem cell meiosis and serves as an inhibitor of transposable elements.^9^ Piwi‐interacting RNAs (piRNAs) are a distinct class of 24 to 32 nucleotide single‐stranded non‐coding RNAs that bind specifically to the Piwi subfamily of Argonaute proteins.^10^ The biogenesis of piRNAs is dicer independent and differs from that of microRNAs. The piRNAs loaded into Piwi proteins play a critical role in epigenetic and post‐transcriptional genetic modification.^11^ In addition to the germline, emerging evidence has recently suggested that piRNAs are present in somatic tissues and some human cancer cells including liver, renal and breast cancers.^12^, ^13^ However, no information on the expression of piRNAs and their role in PH is currently available.

DNA methylation, especially DNA hypermethylation which predominantly occurs on the fifth carbon of cytosine (5mC) in promoters, can frequently result in gene silencing.^14^ This epigenetic modification is controlled by DNA methyltransferase (Dnmts) such as Dnmt1, Dnmt3a and Dnmt3b.^15^ More recently, the whole epigenome‐wide DNA methylation profile of cultured PASMCs from chronic thromboembolic pulmonary hypertension patients and control subjects was analysed. The relationship between PIK3CA DNA methylation and cell proliferation, migration and apoptosis was shown, which could be relevant to pulmonary hypertension.^16^ Archer et al identified the hypermethylation of a CpG island in an enhancer region of superoxide dismutase‐2 (SOD2) as the basis for SOD2 down‐regulation in PH. Demethylation of SOD2 restored mitochondrial function, inhibited PASMC proliferation and increased cell apoptosis.^17^ It is becoming increasingly clear that piRNAs serve as sequence‐specific guides that direct DNA methylation and epigenetic regulation, contributing to the progression of human disorders.^18^, ^19^ These studies led to the hypothesis that piRNAs may be correlated with epigenetic modifications, especially DNA methylation within PASMCs, and may initiate PH pathogenesis. Thus, the present study was designed to investigate the biological role of piRNAs in PH and to further characterize DNA methylation and the potential epigenetic regulatory mechanism of piRNAs in PASMC proliferation.

## 2 MATERIALS AND METHODS

### 2.1 Materials

Antibodies against Piwil1, Piwil2, Stat1, Maoa, Acadm, Dnmt1, Dnmt3a and Dnmt3b were obtained from Abcam (catalogue numbers: ab12337, ab85084, ab92506, ab126751, ab92461, ab188453, ab188470 and ab79822, respectively). PCNA, cyclin A, cyclin D and cyclin E antibodies were obtained from Boster Biological Technology Co. Ltd. (catalogue numbers: BM1582, BM0104, BM4272 and BA0774, respectively). Decitabine, Azacitidine, Bortezomib (PS‐341) and MG‐132 were obtained from Selleck Chemicals (catalogue numbers: S1200, S1782, S1013 and S2619). All other reagents were from common commercial sources.

### 2.2 Small RNA sequencing

Pulmonary arteries (PAs) were rapid separation under a microscope on ice from normoxic and hypoxic rats. Total RNA was extracted, and approximately 10 μg of RNA from each group was separated through 17% denaturing polyacrylamide gels. Small RNA fragments of 18‐28 nt were enriched and purified. RNA adaptors (Illumina) were ligated to the small RNAs followed by reverse transcription into cDNAs. The products were finally subjected to Solexa/Illumina sequencing by SBC (Shanghai Biotechnology Corporation). The CLC Genomics Workbench V5.5 was used to perform piRNA data analysis. The results were screened according to the criterion of significant difference (piRNA expression ≥ 1.5‐fold difference; *P* ≤ .01; FDR ≤ 0.1).

### 2.3 Animals and morphometric analysis

All animal care and experimental procedures were performed in accordance with the National Institutes of Health Guide for the Care and Use of Laboratory animals and were approved by the Institutional Animal Care and Use Committee of Harbin Medical University. About 60 male, 5‐ to 6‐week‐old adult Wistar rats with a mean weight of 120‐150 g were used in this experiment. For establishment of the experimental PH animal models, rats were randomized to treatments of 21 days under normal and hypoxic environments with fractional inspired oxygen (FiO_2_) of 0.21 and 0.12, respectively, as previously described. At the end of the exposure period, the lungs were quickly removed from anaesthetized rats and a portion of the lung tissues was harvested for Western blotting. For haematoxylin and eosin (H&E) staining, the lungs were immersed in 4% paraformaldehyde. The fixed tissues were then embedded in paraffin wax and cut into 5‐μm‐thick sections and stained with haematoxylin and eosin. In situ hybridization was performed using a detection kit (Boster, Wuhan China) in 5‐μm‐thick sections of 4% fixed paraformaldehyde (containing 0.1% diethylpyrocarbonate) of lung tissues. Digoxigenin‐labelled DNA probes complementary to piR‐rno‐63076 were generated using random primer labelling (Boster). The total positive staining area in the vascular walls was quantified in high‐resolution images of individual vessels by image analysis using a colour‐recognition algorithm.

### 2.4 Quantitative RT‐PCR

RNAs were extracted from PAs and PASMCs, respectively, according to the manufacturer's instructions and detected by ultraviolet spectrophotometer. Extracted RNAs were reverse transcribed according to the superscript first‐strand cDNA synthesis kit (Invitrogen). cDNA samples were amplified in a DNA thermal cycler (Bio‐Rad). Conventional real‐time PCR with SYBR Green (Takara) was carried out with total RNA samples as detailed elsewhere.^20^ The nucleotide sequences of primers (5′‐3′) were as follows:

piR‐rno‐63076,GCAGTACCACAGGGTAGA (forward), GGTCCAGTTTTTTTTTTTTTTTCGT (reverse).

piR‐rno‐62974,CAGCAGTGGTTTTACCCTATG (forward), GGTCCAGTTTTTTTTTTTTTTTCTAC (reverse).

RNU6B (U6), GCTTCGGCAGCACATATACTAAAAT (forward), CGCTTCACGAATTTGCGTGTCAT (reverse).

5S rRNA (5S), TACGTGGATGGGAGACCACCT(forward), CAGTTTTTTTTTTTTTTTAAAGCCTACAGC(reverse).

Acadm, AGTCCTTGGCCCCGAATTGT(forward), TCCGCCACATTCCTCAGTGT(reverse).

β‐actin, AGGGAAATCGTGCGTGAC(forward), CAAGAAGGAAGGCTGGAAAA(reverse).

### 2.5 Cell culture and protocols

Briefly, distal PAs were de‐endothelialized and gently digested with collagenase II and 0.1% bovine serum albumin in PBS for 1 hour. The digested PASMCs were then cultured in DMEM supplemented with 15% foetal bovine serum, 1% streptomycin and 1% penicillin for 3‐5 days. PASMCs from passages 2‐5 were used for further experiments. Cells under hypoxic conditions were incubated in a Tri‐Gas Incubator (Heal Force) with a gas mixture containing 92% N_2_, 5% CO_2_ and 3% O_2_ for 24 hours. Before each experiment, the cells were incubated in DMEM without serum overnight to stop growth.

### 2.6 Measurement of right ventricular systolic pressure (RVSP)

The animals were anaesthetized, and the left carotid artery was cannulated for systemic arterial pressure monitoring. After tracheostomy, French Pressure Catheter (Scisense Inc) was connected to the Scisense FA‐404 recorder. When the right jugular vein was exposed, the catheter was inserted into the vein, and finally into the right ventricular vein. RVSP was continuously recorded for 30 minutes.

### 2.7 siRNA design and plasmid construction

Pulmonary arterial smooth muscle cells were transfected with piRNA agomir and antagomir, which were designed and synthesized by GenePharma. Non‐targeted control siRNA (siNC) was used as negative control. The sense sequences were listed below:

piR‐rno‐63076 agomir, sense, 5′‐UACCACAGGGUAGAACCACG‐3′, antisense, 5′‐UGGUUCUACCCUGUGGUAUU‐3′.

piR‐rno‐63076 antagomir, sense, 5′‐CGUGGUUCUACCCUGUGGUA‐3′.

piR‐rno‐62974 agomir, sense, 5′‐CAGUGGUUUUACCCUAUGGUAG‐3′, antisense, 5′‐ACCAUAGGGUAAAACCACUGUU‐3′.

piR‐rno‐62974 antagomir, sense, 5′‐CUACCAUAGGGUAAAACCACUG‐3′.

NC control: sense, 5′‐UUCUCCGAACGUGUCACGUTT‐3′, antisense, 5′‐ACGUGACACGUUCGGAGAATT‐3′.

The Acadm overexpression plasmid was constructed with the GV230 vector by GeneChem. The vector alone was used as a negative control. Transfection was implemented according to the manufacturer's instructions for the Lipofectamine^®^ 2000 reagent (Life Technologies). Then, 2 µg of siRNA and 10 µL of transfection reagent were, respectively, diluted in serum‐free Opti‐MEM‐1 medium and mixed for 20 minutes. After transfection, the cells were quiescent for 24‐48 hours and were used as required.

### 2.8 Western blot analysis

Lung tissues and cultured PASMCs were homogenized with cold lysis buffer. Each sample containing 20‐40 µg protein was separated on 8%‐12% SDS‐PAGE gels and transferred onto nitrocellulose membrane. After blocking with 5% nonfat milk, the protein‐adhered membrane was incubated with specific antibodies against Piwil1 (1:1000), Piwil2 (1:1000), Stat1 (1:1000), Maoa (1:1000), Acadm (1:1000), Dnmt1 (1:1000), Dnmt3a (1:1000), Dnmt3b (1:1000), PCNA (1:1000), cyclin A (1:500), cyclin D (1:500) and cyclin E (1:500) followed by reaction with horseradish peroxidase‐conjugated secondary antibodies and enhanced chemiluminescence reagents imaging.

### 2.9 Cell cycle analysis

Pulmonary arterial smooth muscle cells were treated in groups as indicated and then harvested and fixed using 70% ethanol. DNA fluorescence and flow cytometry were measured using BD FACSCalibur Flow Cytometer. The cells were stained according to the CycleTEST PLUS DNA Reagent Kit protocol. For each sample, 2 × 10^4^ events were accumulated in a histogram. The proportions of cells in the different phases of the cell cycle were calculated from each histogram.

### 2.10 Luciferase assay

Cells (10^5^ per well) were transfected with 1 µg piR‐63076 or 1 µg PGL3‐target DNA (firefly luciferase vector) and 0.1 µg PRL‐TK (TK‐driven Renilla luciferase expression vector), with Lipofectamine 2000, according to the manufacturer's instructions. Luciferase activities were measured 48 hours after transfection with a dual‐luciferase reporter assay kit on a luminometer (Lumat LB9507; dual‐luciferase reporter assay kit, Promega, E1960).

### 2.11 Co‐immunoprecipitation

Cells were lysed in RIPA lysis buffer (Tris 50 mmol/L, pH 7.4, NaCl 150 mmol/L, Triton X‐100 1%, EDTA 1 mmol/L and PMSF 2 mmol/L). A 5 μg target antibody or IgG was added to 0.5 mL of cell lysate and incubated at 4°C for 4‐6 hours. Protein A + G agarose beads were added before the incubation was continued overnight. Antibody‐protein complexes were washed three times with PBS, then the buffer was removed, and the pellet was resuspended in protein loading buffer (2×). The eluted samples were then subjected to Western blot analysis.

### 2.12 DNA extraction and measurement of global DNA methylation levels

Pulmonary arterial smooth muscle cells were collected after transfection or treatment with hypoxia. Total DNA was isolated by TIANamp Genomic DNA Kit (Tiangen). Global DNA methylation levels were assessed by Methylflash™ DNA Methylation (5‐mc) Elisa Kit (Epigentek) according to the manufacturer's protocol.

### 2.13 Bisulfite Sequencing Analysis (BSP)

Bisulfite conversion of genomic DNA was done using the EZ DNA Methylation‐Gold Kit (ZYMO) according to the manufacturer's instructions. Specific fragments were amplified by PCR with 10 μmol/L primers under the following conditions: 94°C for 5 minutes; 10 cycles of 94°C for 30 seconds, 56°C for 30 seconds, and 72°C for 30 seconds; 30 cycles of 94°C for 30 seconds, 50°C for 30 seconds, and 72°C for 30 seconds, followed by an additional incubation at 72°C for 5 minutes. Following PCR amplification, the DNA was extracted from the gel using the DNA gel extraction kit (Tiangen), cloned and transfected into competent bacteria that were grown with ampicillin, 0.2 mm X‐gal, and 0.1 mm isopropyl β‐d‐thiogalactopyranoside. The DNA from positive clones was isolated and sequenced. Obtained sequences were aligned with unconverted genomic DNA sequence using the BiQ Analyzer.

### 2.14 EdU incorporation assay

Pulmonary arterial smooth muscle cells were subjected to different agents and then exposed to 50 μmol/L of 5‐ethynyl‐2’‐deoxyuridine (EdU, RiboBio) for additional 4 hours at 37°C. The cells were fixed with 4% formaldehyde for 15 minutes and treated with 0.5% Triton X‐100 for 20 minutes at room temperature. After washing with phosphate‐buffered saline for three times, the cells were reacted with 100 μl of Apollo^®^ reaction cocktail for 30 minutes. Subsequently, the DNA contents of cells in each well were stained with Hoechst 33342 (5 μg/mL) for 30 minutes and visualized under a fluorescent microscope. The percentage of proliferating cells (EdU‐positive cells) with Hoechst was quantitated using ImageJ software.

### 2.15 RNA‐FISH

Fluorescence‐conjugated piR‐rno‐63076 probes (5′‐CGTGGTTCTACCCTGTGGTA‐3′) were synthesized by (Exon, Guangzhou, China) and used for RNA‐FISH. Hybridization was performed using RNA‐FISH kit (Exon) according to the manufacturer's instructions. Finally, the FISH sections were incubated with DAPI for 10 minutes. And then the images were recorded using a Hamamatsu ORCA‐R2 camera (Hamamatsu Photonics) and recorded by LAS AF software (Leica).

### 2.16 Statistical analysis

Statistical analyses were performed using Prism GraphPad Software (GraphPad Software Inc). Data are presented as means ± SEM. Statistical analysis was performed with Student's *t* test or one‐way ANOVA followed by Tukey's test where appropriate. *P* < .05 was considered statistically significant.

## 3 RESULTS

### 3.1 Altered piRNA expression in PAs under hypoxic conditions

**FIGURE 1:**
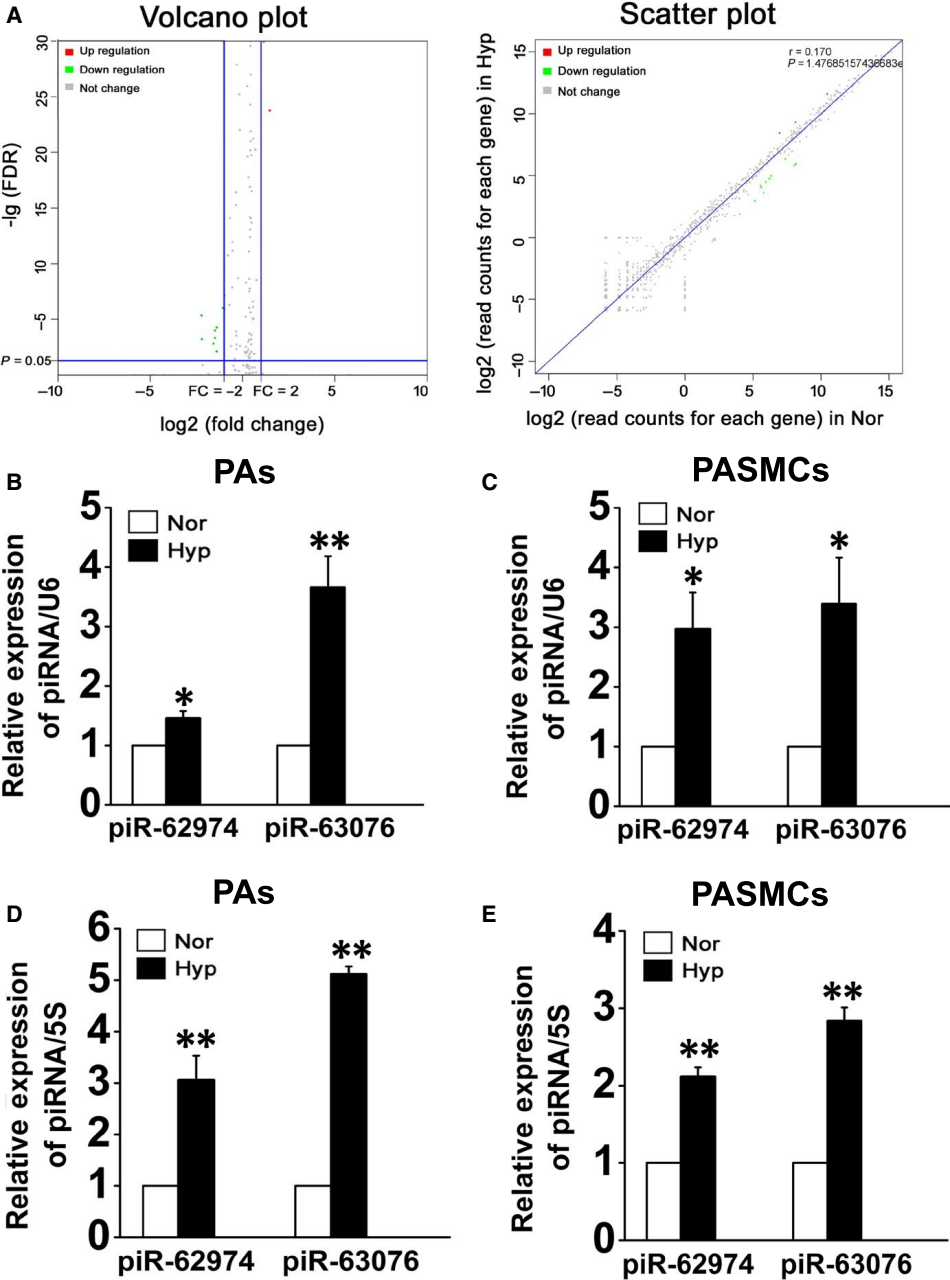
PiR‐62974 and piR‐63076 expression was increased under hypoxic conditions. A, The expression of piRNAs was analysed with small RNA sequencing, and volcano plot and scatter plot showed the whole comparing of normoxic and hypoxic samples’ expression (n = 3). B‐E, qRT‐PCR verification of the expression of up‐regulated piRNAs in PAs from normoxic and hypoxic rats as well as PASMCs cultured under normoxic and hypoxic conditions (B and C, results were normalized to U6. D and E, results were normalized to 5S). *P < .05, **P < .01, (n = 8). All values are denoted as mean ± SEM

The rat models of PH were induced by consecutive hypoxic exposure for 21 days. H&E staining revealed obvious medial thickening of PAs obtained from hypoxic rats (Figure S1A). Moreover, we observed increased right ventricular systolic pressure (RVSP) in rat models of PH after exposed to hypoxia (Figure S1B). The results showed that the hypoxic conditions worked well in PH animal model construction. Then, the expression of piRNAs in pulmonary arteries was analysed by small RNA sequencing. The deep‐sequencing data showed that the major component of the sequence reads was microRNAs. In addition, we found a group of nonannotated sequences with a size of 24‐32 nts, which corresponds to the length of piRNAs. Based on comparison of the sequences with the piRBase database,^21^, ^22^ we confirmed the expression of piRNAs in the hypoxic pulmonary hypertension model for the first time. Volcano and scatter plots were generated to the number of reads for each piRNA between normoxic and hypoxic rats (Figure 1A). In comparison with the normoxic samples, some piRNAs exhibit significant changes in hypoxia. Among these piRNAs, the majority were repressed, and 2 piRNAs were found to be >2‐fold up‐regulated in hypoxic PAs which were piR‐rno‐62974 and piR‐rno‐63076 (Table 1). To confirm the results of deep sequencing, we measured the expression of the identified piRNAs using quantitative real‐time PCR (qRT‐PCR). RNU6B (U6) and 5S rRNA (5S) were used as internal normalizers, respectively. The expression of both piR‐62974 and piR‐63076 was increased in PAs from hypoxic rats and in cultured PASMCs under hypoxia compared to normal conditions (Figure 1B and 1C, values were normalized to U6. Figure 1D and 1E, values were normalized to 5S).

**TABLE 1:** piRNA expression with significant changes in hypoxia

piRNAs	Fold_change‐Hyp/Nor	*P*‐value	FDR	UP/DOWN
piR‐rno‐62935	0.215956	9.84E‐36	7.46E‐34	DOWN
piR‐rno‐62980	0.216681	1.67E‐35	1.18E‐33	DOWN
piR‐rno‐63174	0.216913	1.62E‐35	1.19E‐33	DOWN
piR‐rno‐62944	0.217239	5.61E‐33	3.72E‐31	DOWN
piR‐rno‐62937	0.218448	1.68E‐07	4.30E‐06	DOWN
piR‐rno‐62946	0.221002	2.54E‐05	0.000574649	DOWN
piR‐rno‐62912	0.340386	6.74E‐05	0.001459269	DOWN
piR‐rno‐62883	0.362288	4.03E‐06	9.71E‐05	DOWN
piR‐rno‐62898	0.363258	1.88E‐05	0.000428145	DOWN
piR‐rno‐63029	0.386488	0.000357875	0.00716762	DOWN
piR‐rno‐57218	0.387354	2.16E‐06	5.27E‐05	DOWN
piR‐rno‐63076	2.229279	9.89E‐150	4.20E‐147	UP
piR‐rno‐62974	2.824898	3.14E‐26	1.63E‐24	UP

### 3.2 piRNAs are involved in hypoxia‐mediated PASMC proliferation and cell cycle progression

**FIGURE 2:**
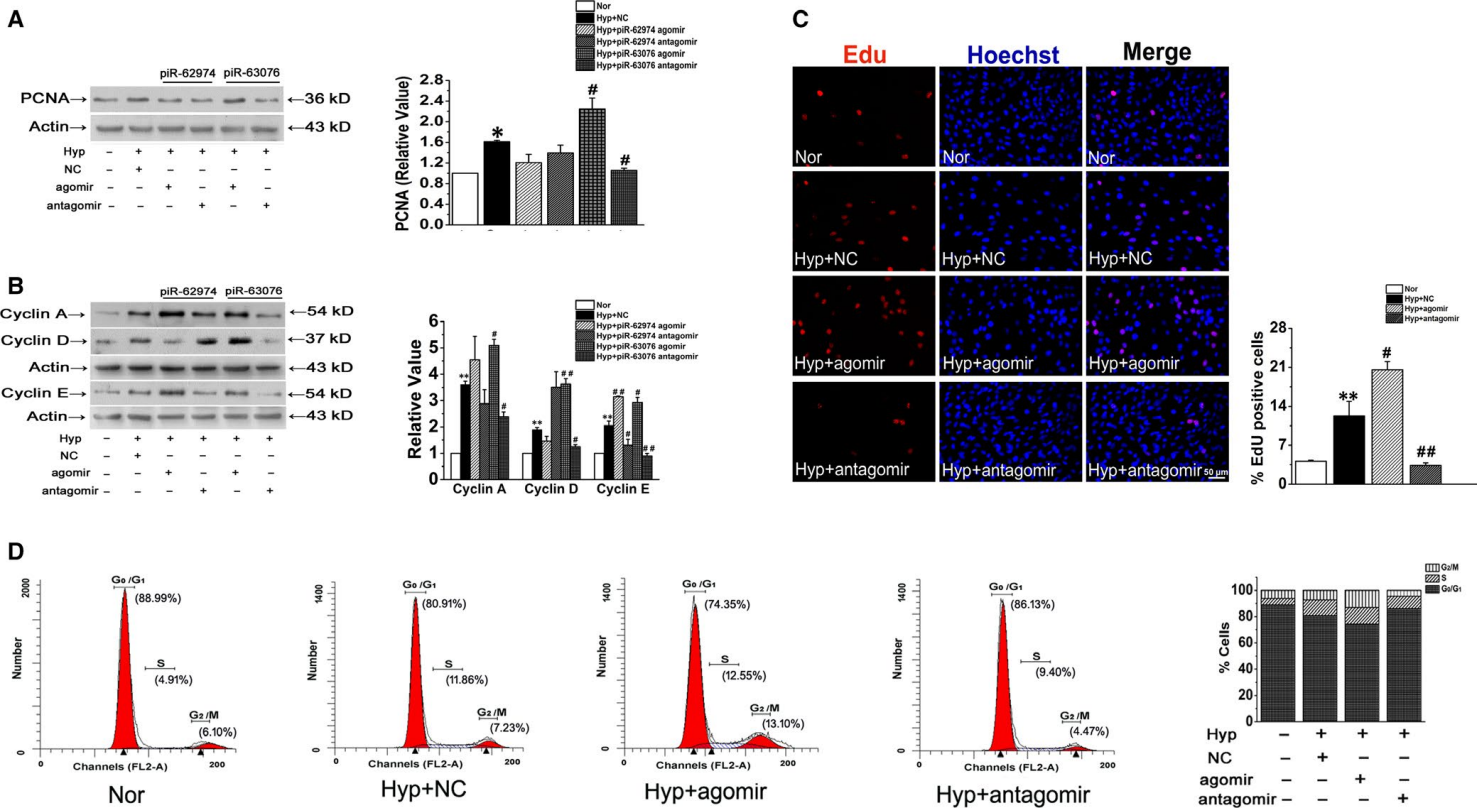
PiR‐63076 mediated hypoxia‐induced PASMC proliferation but not piR‐62974. A, The expression of PCNA was increased in hypoxia which was magnified by piR‐63076 agomir and reversed by piR‐63076 antagomir. B, The protein expression of cyclin A, cyclin D and cyclin E in PASMCs under different conditions. C, Fluorescent EdU incorporation (red) co‐stained with Hoechst (blue to demonstrate nuclear) proved that hypoxia‐induced DNA synthesis was blocked by piR‐63076 inhibitor. Scale bar = 50 μm. D, Fluorescence‐activated cell sorting analyses detected the number of cells in each phase of the cell cycle in PASMCs. *P < .05, **P < .01 compared with normoxia. ^#^ *P* < .05, ^##^ *P* < .01 compared with hypoxia and NC. n = 6. All the values are denoted as mean ± SEM

To further confirm the roles of piR‐62974 and piR‐63076 in PASMC homeostasis, double‐stranded agomir (piRNA mimic) and antagomir (antisense oligonucleotide) complementing the mature piRNAs were used to overexpress or inhibit piRNAs. The transfection efficiency of agomir and anataomir on expression of piRNAs was demonstrated in Figure S1C. Then, the effect of the piRNAs on hypoxic cell proliferation was measured by evaluating the expression of proliferating cell nuclear antigen (PCNA) and cyclins. Figure 2A showed that transfection with the piR‐63076 agomir significantly increased the proliferation of PASMCs, whereas the piR‐63076 antagomir inhibited PCNA expression under hypoxic conditions. However, there was no difference between the effects of the piR‐62974 agomir/antagomir and hypoxia on PCNA expression. Similarly, hypoxia increased the expression of cyclin A, cyclin D and cyclin E, and the effect was promoted in the presence of piR‐63076 mimics and reduced by the piR‐63076 inhibitor. Additionally, only the expression of cyclin E was increased by the piR‐62974 agomir and decreased by the piR‐62974 antagomir (Figure 2B). These results suggested that piR‐63076 was able to regulate PASMC proliferation but piR‐62974 was not. Next, the EdU (5‐ethynyl‐2’‐deoxyuridine) incorporation assay was performed. The percentage of proliferating cells (EdU‐positive cells) was found to be increased under hypoxia, which was decreased by piR‐63076 silencing (piR‐63076 antagomir). The hypoxia‐stimulated EdU incorporation was further increased by the piR‐63076 agomir (Figure 2C). Evaluation of the effect of the piR‐63076 on cell cycle progression showed that piR‐63076 agomir accelerated the S and G_2_/M phase of cell cycle progression under hypoxic conditions, accompanied by a reduction in the number of cells in the G_0_/G_1_ phase compared with cells treated with the negative control (Figure 2D). These results confirm that piR‐63076 plays an important role in regulation of cell cycle progression during hypoxia.

### 3.3 The characterization of piR‐63076

**FIGURE 3:**
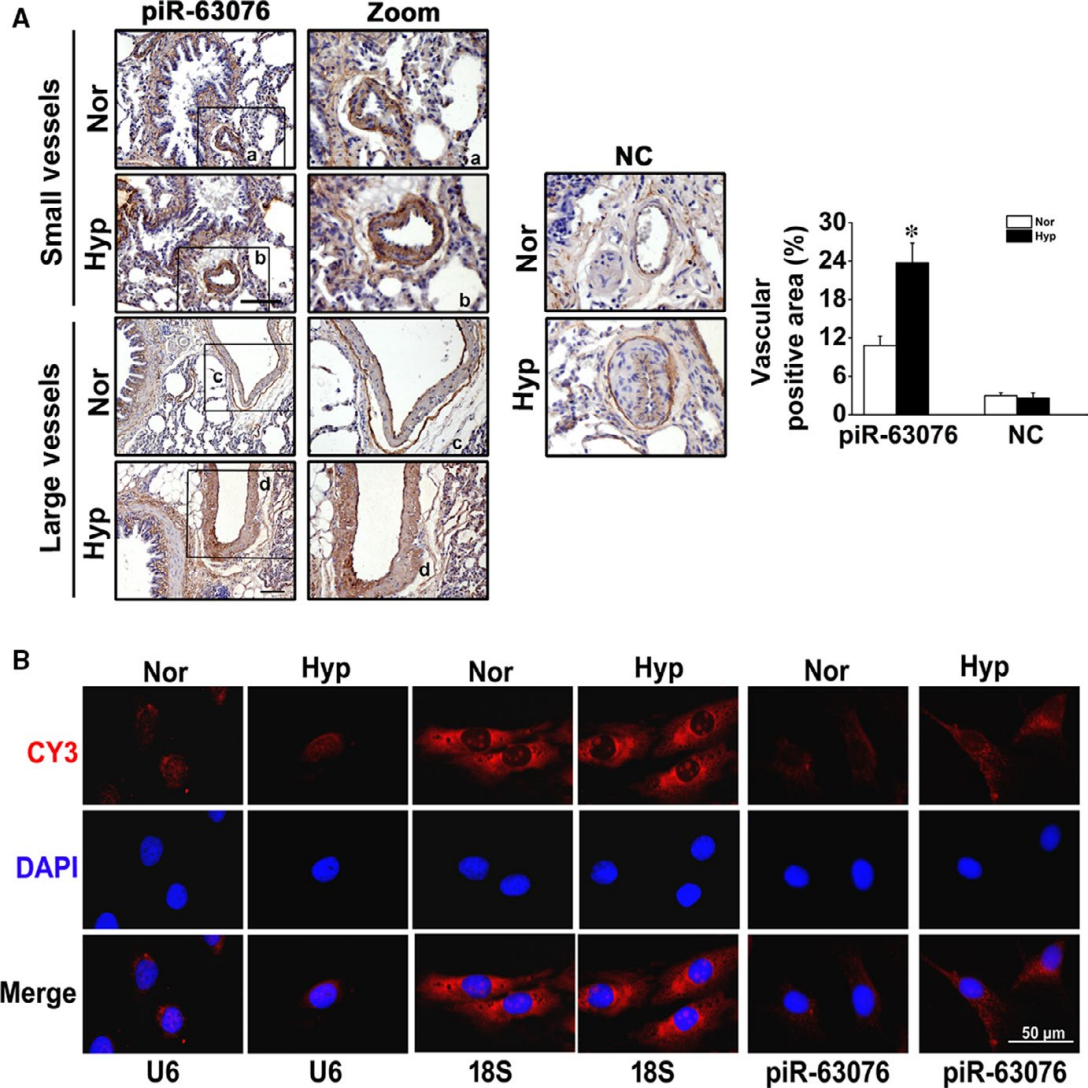
In situ hybridization and RNA‐fluorescent in situ hybridization (FISH) assay showed the distribution of piR‐63076 under hypoxic conditions. A, Localization and quantitative analysis of positive staining per vascular area of piR‐63076 and NC expression in lung tissue. B, FISH assay was performed to detect piR‐63076 expression in cultured cells. U6 and 18S RNA were used as controls for localization of the nucleus and cytoplasm. Scale bar = 50 μm. *P < .05. n = 4. All values are denoted as mean ± SEM

Moreover, the precise location of piR‐63076 in vivo was examined by in situ hybridization methods, and brown and yellow colours indicated the positive stains. High expression of piR‐63076 was observed in hypoxic PAs and was mainly localized in the vascular medial region, whereas in normal lung tissue, piR‐63076 was less detectable, and there was little staining for the NC probe with no difference between the two groups (Figure 3A). The expression of piR‐63076 in PASMCs as shown in Figure 3B was found to be distributed in both the cytoplasm and nucleus using U6 and 18S staining for nuclear and cytoplasmic areas, respectively. Expression of U6 and 18S RNA was not changed by hypoxic exposure. PASMCs cultured under hypoxic conditions were found to have higher piR‐63076 levels compared to those cultured under normoxic conditions. Furthermore, UCSC Genome Browser image in Figure S2A showed that the sequence of piR‐63076 is conserved across 20 different species. According to the piRBase database, the genomic localization of piR‐63076 is at chr19:39,609,010‐39,609,030. The detailed chromosome information from Ensembl was shown in Figure S2B.

### 3.4 PiR‐63076 regulates Acadm expression in PASMCs

**FIGURE 4:**
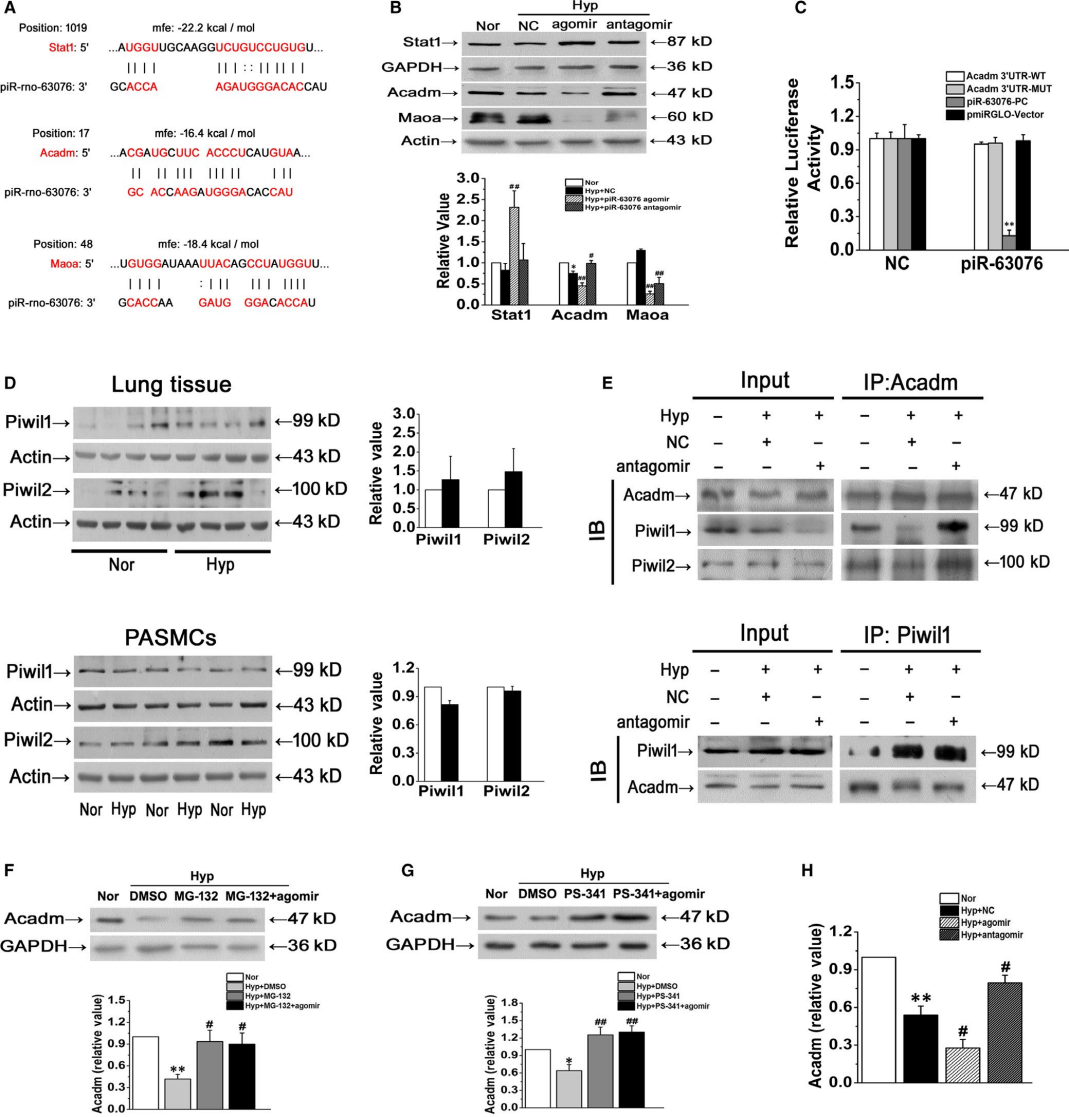
Acadm is the targets of piR‐63076 and cooperated with Piwi proteins. A, Computational analysis was performed for the complementarities of piR‐63076 sequence to the target mRNAs. B, Western blot assay in PASMCs transfected with scrambled piRNA (NC) and cholesterol, methoxy and thiosulfate modified piR‐63076 mimics (agomir) and piR‐63076 inhibitors (antagomir) (n = 6). C, Luciferase assay for piR‐63076 and Acadm combination. HEK‐293 cells were cotransfected with scrambled piRNA (NC) and Acadm 3’‐UTR (WT)/ mutant Acadm 3’‐UTR (MUT)/ complementary sequences of piR‐63076 (piR‐63076‐PC)/ Vector (n = 3). D, Pulmonary expression of Piwi proteins (Piwil1 and Piwil2) was induced by hypoxia in vivo and in vitro (n = 6). E, PASMCs were exposed to different treatment for 24 h, and the whole cell lysates were extracted for co‐immunoprecipitation with anti‐Acadm or anti‐Piwi proteins, followed by probing with anti‐Piwil1 or anti‐Acadm, respectively (n = 3). F and G, Immunoblotting showing that addition of MG‐132 and PS‐341 blocked down‐regulation of Acadm induced by hypoxia in PASMCs, piR‐63076 failed to regulate Acadm expression after MG‐132 and PS‐341 treatment (n = 4). H, qPCR analyses showing that overexpression of piR‐63076 diminished the level of Acadm mRNA (n = 4). Nor: normoxia; Hyp: hypoxia. *P < .05, **P < .01 compared with normoxia, ^#^ *P < *.05, ^##^ *P* < .01 compared with hypoxia. All of the values are denoted as mean ± SEM

The above observations showed that piR‐63076 was involved in hypoxia‐induced aberrant PASMC proliferation. It is possible that these actions of piR‐63076 resulted from the regulation of distinct targets related to proliferative factors. We predicted target proteins that were associated with piR‐63076 by computation and a bioinformatics‐based approach employing the miRanda program with the default parameters and cut‐offs (Score S ≥ 140 and Energy E ≤ –7.0) and using the RNAhybrid database as previously described.^23^ These analyses led to the identification of 3 candidate targets of piR‐63076: signal transducer and activator of transcription 1 (Stat1), acyl‐CoA dehydrogenase (Acadm) and monoamine oxidase A (Maoa) (Figure 4A). To prove that Stat1, Acadm and Maoa are indeed repressed post‐transcriptionally by piR‐63076, we determined the effect of piR‐63076 on protein expression. Figure 4B shows that the protein levels of Acadm were decreased coinciding with the up‐regulation of piR‐63076 and that inhibition of piR‐63076 with antagomir increased the expression of Acadm. At the same time, the changes in Stat1 and Maoa were irregular under hypoxia with or without piR‐63076 interference. To verify whether piR‐63076 directly targets the 3’‐UTR of Acadm, a luciferase assay was performed using 3’‐UTR sequence fragments containing the predicted target region of piR‐63076 and its mutated version, which was inserted downstream of a luciferase reporter. Unexpectedly, the results showed that there was no difference in luciferase activity under piR‐63076 cotransfection with Acadm 3’‐UTR‐WT compared with Acadm 3’‐UTR‐mutated (MUT) (Figure 4C). The complementary sequence of piR‐63076 (piR‐63076‐PC) was set as a positive control. This result implied that the molecular mechanism of the effect of piR‐63076 on the target gene may be different from that of microRNAs by acting on the 3’‐UTR. To fully reveal the detailed mechanism between piR‐63076 and the reduction of Acadm, we then detected the expression of Piwi proteins, which serve as functional complexes interacting with piRNAs. We observed expression of Piwil1 and Piwil2 in lung tissues and in cultured PASMCs for the first time. However, the expression of the two Piwi proteins was no further changes after hypoxia treatment (Figure 4D). Moreover, co‐immunoprecipitation assay demonstrated that there was an interaction between Piwi proteins and Acadm (Figure 4E). These results indicate that piR‐63076 is responsible for the regulation of Acadm in hypoxia independent of microRNA action. We next assessed if piR‐63076 down‐regulates Acadm through a proteasome‐dependent degradation pathway. To this end, we treated cells with proteasome inhibitors MG132 (carbobenzoxyl‐L‐leucyl‐L‐leucyl‐L‐leucinal) or PS‐341 (Bortezomib). Subsequent Western blot analysis showed a decreased expression of Acadm in hypoxia, which was reversed by MG132, and the process is not changed after piR‐63076 agomir transfection (Figure 4F). Similar results were observed in PS‐341 treated cells, suggesting that the down‐regulation of Acadm in PASMCs mediated by piR‐63076 is in a proteasome‐independent manner (Figure 4G). To test if piR‐63076 regulates Acadm expression transcriptionally, we measured the mRNA expression of Acadm. Representative results showed that piR‐63076 agomir further reduced the level of Acadm mRNA compared to hypoxia, while the mRNA expression of Acadm was increased when piR‐63076 was depleted by antagomir (Figure 4H).

### 3.5 PiR‐63076 abrogation reduces the methylation status of the Acadm promoter

**FIGURE 5:**
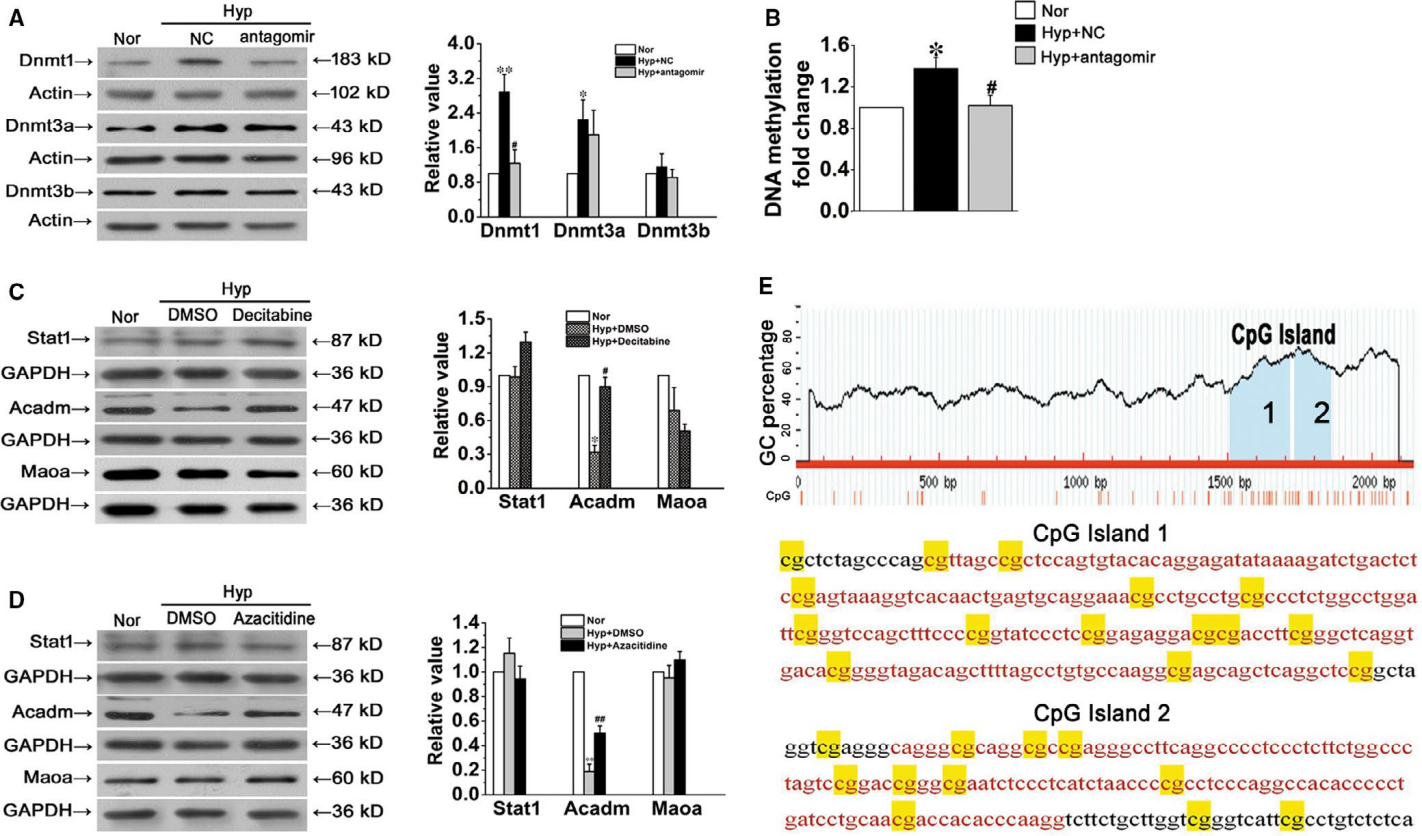
The association between piRNA‐63076 and methylation. A, The protein expression levels of Dnmt1, Dnmt 3a and Dnmt 3b. B, Global DNA methylation changes induced by piR‐63076 in PASMCs was measured after hypoxia and transfection. C and D, Demethylation by decitabine and azacitidine increases the expression of Acadm in hypoxia, but not Stat1 and Maoa. E, Schematic of the CpG islands within the Acadm promoter. Nor, normoxia; Hyp, hypoxia. *P < .05, **P < .01 compared with normoxia. ^#^ *P* < .05, ^##^ *P* < .01 compared with hypoxia plus NC. n = 6. All of the values are denoted as mean ± SEM

**FIGURE 6:**
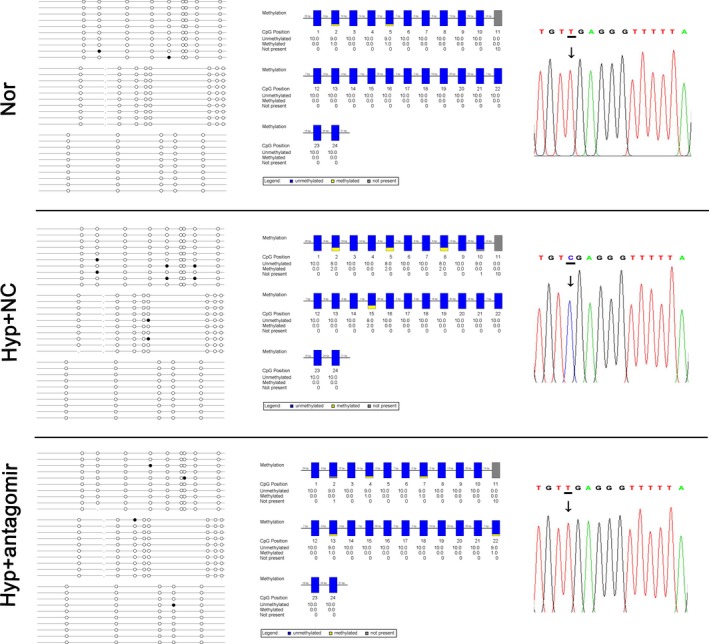
Mapping of the BSP results in the Acadm promoter region. The filled circles represent methylated CG sites, and hollow circles represent unmethylated CG sites (the left panel). Yellow indicates methylated position, blue indicates unmethylated position, and grey indicates no detected CG sites (the middle panel). A short representative sequence of sequencing traces from the Acadm promoter region in cultured PASMCs. Only methylated cytidines are protected against bisulfite‐mediated deamination of cytidine into uridine (thymidine in the amplified PCR product) (the right panel)

The above results suggest that piR‐63076 regulated expression of Acadm in hypoxia may be partly achieved through influences of the Acadm mRNA level. As DNA methylation is one of the most common modifications of the transcription level, and emerging evidence has suggested that the Piwi/piRNA complex may play an epigenetic silencing role on target genes through DNA methylation.^24^ To evaluate the significance of DNA methylation in hypoxia, the expression of DNA methyltransferases, including Dnmt1, Dnmt3a and Dnmt3b, was first measured. As shown in Figure 5A, the expression of Dnmt1 and Dnmt3a was up‐regulated in hypoxia compared with normoxia. Moreover, the increase in response to hypoxia of Dnmt1 was particularly down‐regulated followed by piRNA‐63076 inhibition. Similarly, increased global DNA methylation, as measured by the 5mC incorporation assay, was found in hypoxic cells which were decreased by antagomir‐piRNA‐63076 treatment (Figure 5B). To explore whether the expression of piR‐63076 target genes is associated with DNA methylation, cells were treated with two different inhibitors of methylation: the demethylation agents 5‐aza‐2’‐deoxycytidine (decitabine) and azacitidine, under hypoxia. Western blot analysis demonstrated that the decrease in Acadm expression observed under hypoxia was successfully recovered by decitabine and azacitidine treatment, whereas the expression of Stat1 and Maoa remained insignificant in PASMCs (Figure 5C,D). Accordingly, analysis using Methyl Primer Express (v1.0), Figure 5E revealed the presence of two CpG islands (positions 1506‐1716 and 1731‐1859) in the Acadm gene promoter region. To further address the role of piRNA‐63076 in the epigenetic modifications of Acadm, we analysed the methylation status in the promoter region of Acadm by using bisulfite sequencing PCR (BSP). The Acadm PCR products were then sequenced, which demonstrated increased methylation at the Acadm promoter region in hypoxia, while lower methylation was found in the BSP product of antagomir‐piRNA‐63076‐transfected cells (Figure 6). These experiments indicate that one probable reason for the piRNA‐63076‐induced inhibition of Acadm in hypoxia is DNA methylation of the Acadm promoter region.

### 3.6 Acadm deficiency increases hypoxia‐induced cell proliferation

**FIGURE 7:**
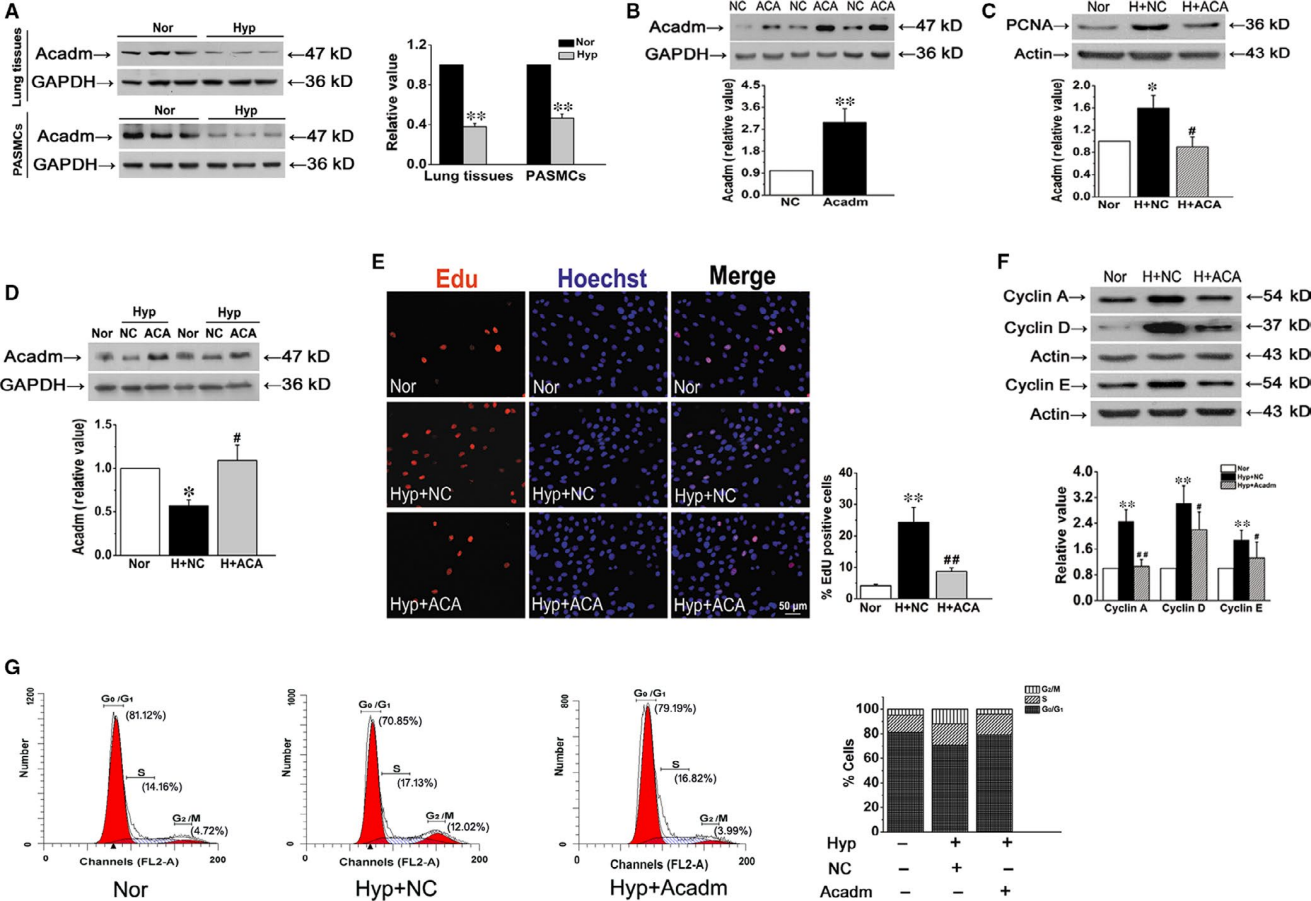
Effect of Acadm on PASMC proliferation in hypoxia. A, The expression of Acadm in vivo and in vitro was detected by Western blot. B, Protein expression of intracellular Acadm was measured to assess the efficiency and specificity of plasmid targeting Acadm overexpression. C, The protein expression of PCNA was determined by Western blot analysis. D, Acadm protein overexpression was measured by Western blot in hypoxia. E, Proliferation of PASMCs was evaluated by EdU incorporation assay. Scale bar = 50 μm. F, The protein expression levels of cyclin A, cyclin D and cyclin E. G, The number of cells in each phase of the cell cycle was examined by flow cytometry in normoxia or hypoxia. Nor, normoxia; H and Hyp, hypoxia; ACA, Acadm overexpression. *P < .05, **P < .01 compared with normoxia. ^#^ *P* < .05, ^##^ *P* < .01 compared with hypoxia plus NC. n = 6. All of the values are denoted as mean ± SEM

Next, Acadm expression in lung tissues and PASMCs was evaluated. As expected, minimal Acadm expression was found in both lung tissues and PASMCs under hypoxic condition (Figure 7A). However, the role of Acadm in regulating PASMC function has not been demonstrated. To address this issue, we transfected cultured PASMCs with a plasmid overexpressing Acadm. Acadm protein expression was up‐regulated approximately threefold in plasmid‐transfected cells compared with the negative control (Figure 7B). Then, the expression of PCNA was detected, hypoxia increased the expression of PCNA, and the effect was reserved by overexpressing Acadm (Figure 7C). Further, to ensure the effect of transfection in various analyses, overexpression of Acadm in hypoxia was also detected. Figure 7D confirmed that Acadm overexpression was valid in hypoxic PASMCs. After that, the percentage of EdU‐positive cells was found to be increased under hypoxia, which was reduced by Acadm overexpression (Figure 7E). We observed a similar trend when we evaluated the post‐translational levels of the cell cycle‐related proteins cyclin A, cyclin D and cyclin E in cultured PASMCs (Figure 7F). Assessment of the effect of Acadm on cell cycle progression showed that overexpression of Acadm decreased the percentage of G_2_/M + S phase cells under hypoxic conditions from 29.15% to 20.81%, accompanied by an increase in cells in the G_0_/G_1_ phase from 70.85% to 79.19% (Figure 7G). These results suggested that Acadm recovery led to the decreased PASMCs proliferative capability observed under hypoxia.

## 4 DISCUSSION

The major finding of this study was the identification of piRNAs as a potential new epigenetic mechanism involved in PH in hypoxic animal model. More specifically, we initially found that piRNA‐63076 was up‐regulated in hypoxic experimental PH models and cultured PASMCs by performing deep sequencing of small RNAs. The consequences of piRNA‐63076 enrichment contributed to PASMC homeostasis disorders, including accelerated cell cycle progression under hypoxic conditions. The downstream target of piRNA‐63076, Acadm, is involved in hypoxia‐induced aberrant cell proliferation. Furthermore, piRNA‐63076 mediates Acadm expression by regulating promoter DNA methylation.

The discovery that piRNAs exist outside the germline and are present in several major somatic tissues, the nervous system and some human cancer cells suggests greater functional significance for piRNAs than was previously appreciated.^25^, ^26^ For instance, by deep sequencing of the small RNA transcriptome, piR‐Hep1 was found to be up‐regulated in hepatocellular carcinoma tumours compared to matched non‐cancerous tissues.^27^ In addition, up‐regulation of piR‐651 has been proposed to be associated with gastric cancer carcinogenesis by enhancing cell proliferation and suppressing cell apoptosis.^28^ Chu et al found that the expression of the Piwil1 protein in bladder cancer tissues was lower than in adjacent normal bladder tissues and identified a novel piR‐ABC associated with bladder cancer via the target TNFSF4 gene.^29^ However, the role of piRNAs in PH and the potential mechanisms of the Piwi/piRNA complex in PASMCs remained undiscovered prior to this work. In this study, we found that piR‐62974 and piR‐63076 are up‐regulated by hypoxia and that piR‐63076 promotes cell proliferation and specifically affects the cell cycle progression of PASMCs. We speculate that piR‐63076 is not the only piRNA affecting pulmonary vascular function. Further basic studies are urgently needed to determine the global piRNA expression profile in PH and to address the possibility that more piRNAs may function as therapeutic targets in PASMC proliferation. Additionally, it is necessary to investigate how Piwi proteins and the Piwi/piRNA complex are involved in biological functions under PH, such as transposon silencing, epigenetic regulation, transcriptional activity and chromosome condensation.

Bioinformatics‐based analysis was used for predicting physical interactions between putative sites of potential targets with piR‐63076. Several target genes were suggested, including Maoa, Stat1 and Acadm. Our data showed that only the expression of Acadm was decreased by the piR‐63076 agomir and increased by the piR‐63076 antagomir. These results suggest that Acadm is a direct target of piR‐63076 but Maoa and Stat1 are not. Acadm catalyses the initial dehydrogenation of fatty acyl‐CoA esters, which is a key step in mitochondrial fatty acid β‐oxidation.^30^ Patients with Acadm deficiency suffer from primary and lethal metabolic attacks in infancy.^31^ Acadm deficiency was previously shown to be associated with heart failure and acute lung injury through the accumulation of fatty acid intermediates such as octanoic and decanoic acids, and to induce oxidative stress.^32^, ^33^, ^34^ To investigate whether Acadm may participate in the pathogenesis and progression of PH, we first showed that Acadm expression was inhibited in hypoxic PASMCs. Additionally, the introduction of an Acadm overexpression plasmid substantially rescued the acceleration of DNA synthesis and slowed PASMC proliferation. A recent study showed that piRNAs act as miRNAs to induce mRNA deadenylation and decay.^35^ Interestingly, a luciferase reporter assay showed that luciferase activity did not differ under the cotransfection of piR‐63076 mimic with either the Acadm3’‐UTR or the Acadm 3’‐UTR with binding site mutation. These results lead us to speculate that the mechanism whereby piR‐63076 regulates gene expression is not by binding to conserved regions in the 3’‐UTR of target genes and suppressing either the translation or mRNA stability of genes, thus differing from the mechanism of action of microRNAs.

To investigate the possible mechanism of Acadm reduction in hypoxia, we therefore analysed the expression of the other Argonaute family protein‐Piwi proteins in lung tissues and in cultured PASMCs after hypoxic treatment. Our results revealed that both Piwil1 and Piwil2 existed outside the germline in lungs, although the expression level was unchanged. It is noteworthy that the Piwi proteins have been implicated in transcriptional gene silencing through directed DNA methylation and histone modifications.^36^, ^37^ For instance, murine Piwi orthologMili and Miwi2 were reported to act as the upstream of Dnmt3l, a DNA methyltransferase enzyme, and were required for de novo methylation of transposon sites.^38^ Additionally, a recent study showed that piR‐31470 as a sequence‐guidance molecule to recognize transcripts of glutathioneS‐transferase pi 1 (GSTP1) and bind to Piwil4 to form the Piwil4/piR‐31470 complex.^39^ The complex contributed to the recruitment of Dnmt1 and Dnmt3α to induce the methylation the CpG island of GSTP1.^39^ Importantly, in our study, we found that Acadm could interact with Piwi proteins, which suggested that Piwi proteins might participate in the piR‐63076 pathway to regulate the expression of Acadm. It is conceivable that a mechanism exists which allows piR‐63076 to guide Piwi proteins and activate epigenetic factors such as DNA methyltransferase enzymes to form a piRNA‐induced silencing complex for targeting Acadm.

It has been demonstrated that the Piwi‐piRNA pathway plays a pivotal role as a specificity determinant of de novo DNA methylation in germ cells.^18^ In multiple myeloma, piRNA‐823 exhibits a positive correlation with Dnmt3a/3b, directly participating in regulating the aberrant DNA methylation of gene promoter regions to inactivate putative tumour‐suppressor genes.^40^ In the present study, decitabine and azacitidine, two methyltransferase inhibitors, increased Acadm expression by demethylating Acadm in PASMCs. PiR‐63076 inhibition led to decreased levels of Dnmt1 expression, with consequent reexpression of Acadm. Moreover, we identified two CpG islands (211 bp and 129 bp) in the promoter region of Acadm. CpG islands have been universally reported to be the regions responsible for the transcriptional repression of genes by methylcytosine.^41^ The presence of CG methylation in the amplified Acadm promoter region (<5% methylation, approximately), while differing from the high percentage of methylation observed for oncogenes, nevertheless reveals a close correlation of DNA methylation with Acadm silencing. To our knowledge, this is the first time that a specific piRNA has been observed to affect the methylation profile of PASMCs, expanding the epigenetic regulatory role of piRNAs to PH.

In conclusion, our study demonstrated that piR‐63076 was up‐regulated in pulmonary vessels and promoted cell proliferation by enhancing cell cycle progression in PASMCs. In addition, the molecular mechanism by which piR‐63076 exerted its proliferative function involved the interaction with Acadm to promote its methylation at adjacent CpG sites, along with changes in transcriptional activity, inducing the degradation of Acadm. Our data suggest that the Piwi/piRNA complex may be a new class of molecules that are potentially useful as biomarkers for the classification of pulmonary hypertension as well as therapeutic targets.

## CONFLICT OF INTEREST

The authors confirm that there are no conflicts of interest.

## AUTHOR CONTRIBUTIONS

DZ designed research; CM and LZ analysed data; XW, SH, JB, CZ, MZ, JZ, WX and Y. L performed research; CM wrote the paper; and QL and XY contributed new reagents or analytic tools. All authors read and approved the final manuscript.

## Supporting information

Figure S1

Figure S2

## Data Availability

The data that support the findings of this study are available from the corresponding author upon reasonable request.
