# Supplementary figures and images for: piRNA‐63076 contributes to pulmonary arterial smooth muscle cell proliferation through acyl‐CoA dehydrogenase
Source: J Cell Mol Med. 2020 Mar 30;24(9):5260–73. doi: 10.1111/jcmm.15179 (PMC7205801; doi:10.1111/jcmm.15179)

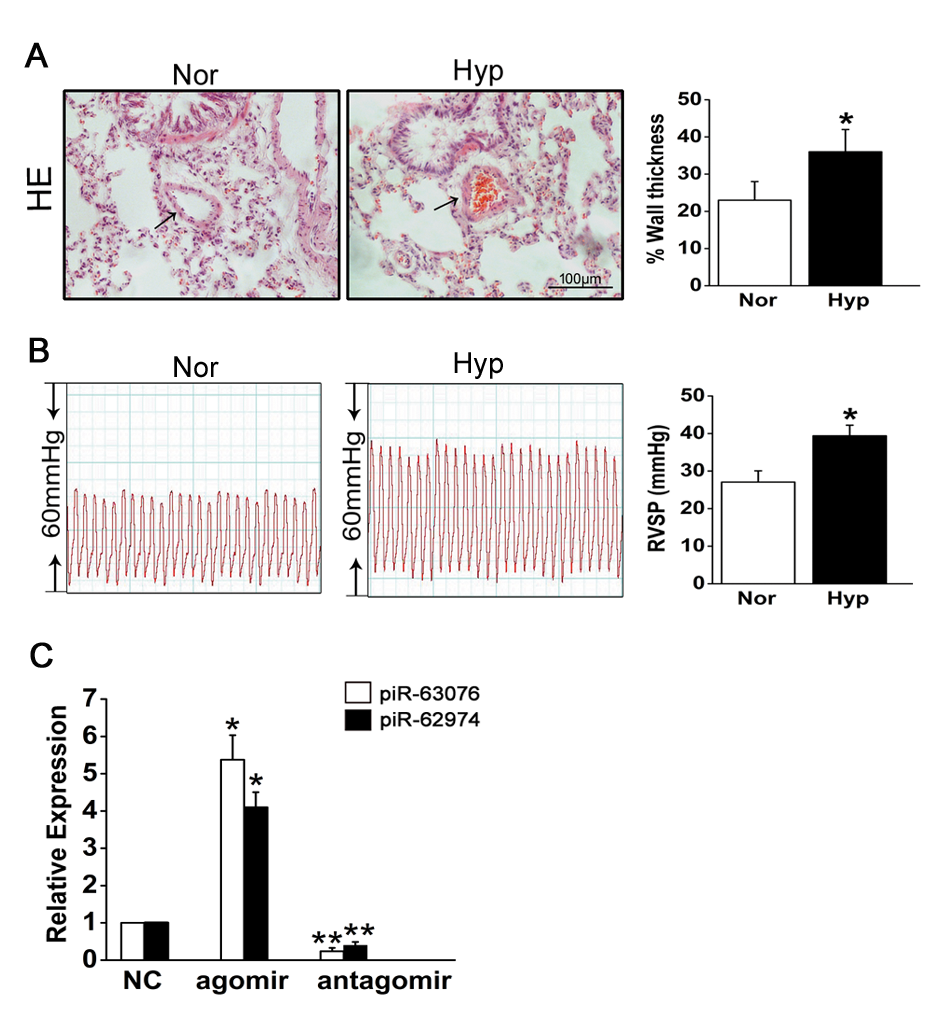

Supplement: Supplementary file 1 — Figure S1 [file JCMM-24-5260-s001.tif]

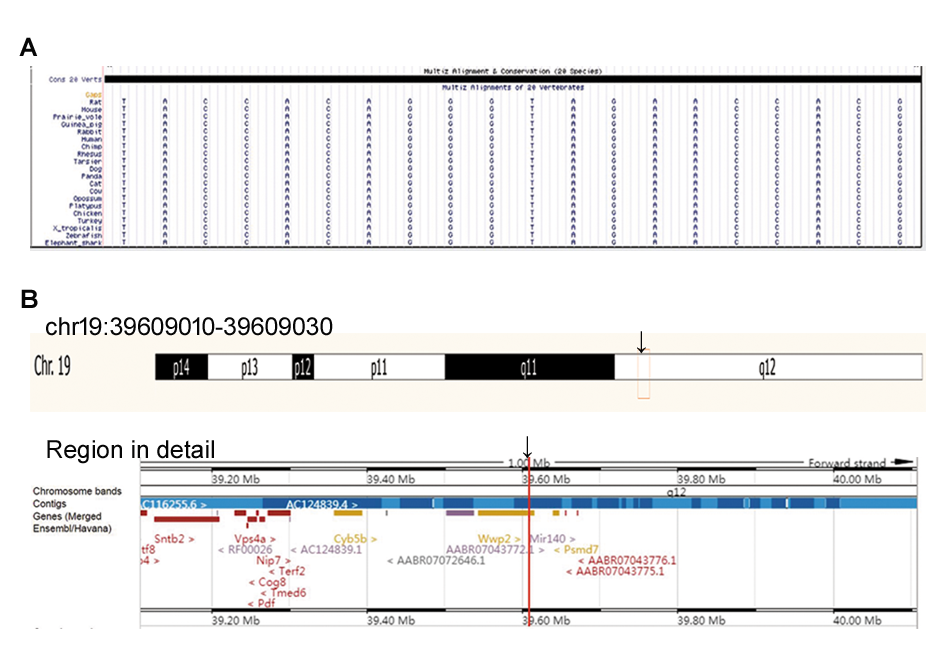

Supplement: Supplementary file 2 — Figure S2 [file JCMM-24-5260-s002.tif]
